# Dexmedetomidine inhibits neuronal apoptosis by inducing Sigma-1 receptor signaling in cerebral ischemia-reperfusion injury

**DOI:** 10.18632/aging.102404

**Published:** 2019-11-04

**Authors:** Meili Zhai, Chong Liu, Yuexiang Li, Peijun Zhang, Zhiqiang Yu, He Zhu, Li Zhang, Qian Zhang, Jianbo Wang, Jinhua Wang

**Affiliations:** 1 Department of Anesthesiology, Tianjin Central Hospital of Gynecology Obstetrics, Gynecology Obstetrics Hospital of Nankai University, Tianjin Key Laboratory of Human Development and Reproductive Regulation, Tianjin 300052, China; 2 Department of Anesthesiology, Central Laboratory, Tianjin 4th Centre Hospital, The Fourth Central Hospital Affiliated to Nankai University, Tianjin 300140, China; 3 Department of Anesthesiology, Tianjin Xiqing Hospital, Tianjin 300380, China; 4 Department of Neurology, Taizhou Central Hospital (Taizhou University Hospital), Taizhou, Zhejiang Province 318000, China

**Keywords:** dexmedetomidine, apoptosis, cerebral ischemia-reperfusion injury, Sigma-1 receptor

## Abstract

Dexmedetomidine is known to alleviate cerebral ischemia-reperfusion injury (CIRI). We established a rat model of CIRI, which exhibited higher neurological deficit scores and a greater number of apoptotic cells in the cerebral ischemic penumbra than controls. Dexmedetomidine reversed the neuronal apoptosis and improved neurological function in this model. We then examined Sigma-1 receptor (Sig-1R) expression on the endoplasmic reticulum (ER) in brain tissues at different reperfusion time points. Sig-1R expression increased with CIRI and decreased with increasing reperfusion times. After 24 hours of reperfusion, dexmedetomidine upregulated Sig-1R expression, and ER stress proteins (GRP78, CHOP, JNK and Caspase-3) were detected in brain tissues with Western blotting. Moreover, GRP78 expression followed a pattern similar to Sig-1R. Dexmedetomidine induced GRP78 expression but inhibited CHOP, Caspase-3 and phosphorylated-JNK expression in brain tissues. A Sig-1R-specific inhibitor reduced GRP78 expression and partially inhibited the upregulation of GRP78 by dexmedetomidine. The inhibitor also increased CHOP and Caspase-3 expression and partially reversed the inhibitory effects of dexmedetomidine on these pro-apoptotic ER stress proteins. These results suggest that dexmedetomidine at least partially inhibits ER stress-induced apoptosis by activating Sig-1R, thereby attenuating brain damage after 24 hours of ischemia-reperfusion.

## INTRODUCTION

Cerebral infarction, also known as ischemic stroke, is the most common neurological disease. In recent years, the incidence of cerebral infarction has been increasing, creating an enormous burden for families and society. Despite extensive research, no effective treatment has been found for this common disease [1].

When ischemic stroke occurs, the tissues are damaged to varying degrees, and the damage is often irreversible. After a period of ischemia, the blood supply to the brain gradually recovers, but a large number of nerve cells fail to return to normal function in time. This can cause more serious neurological damage, in what is called cerebral ischemia-reperfusion injury (CIRI) [2]. CIRI leads to neuronal death in the core area of the occlusive artery within a few minutes, while a certain number of surviving but vulnerable neurons form an ischemic penumbra around the core area [3].

Due to collateral circulation, neurons in the ischemic penumbra undergo less damage than those in the core area, and are able to maintain a normal ion balance and structural integrity, which enables them to survive for hours or longer; thus, these cells have become a focus of research on CIRI and brain protection. Improvements in treatment have made it possible to open the occluded cerebral blood vessels more rapidly and restore the blood flow in the ischemic penumbra in patients with cerebral infarction, resulting in a very good prognosis for some patients [4, 5]. However, due to the shortness of the effective treatment window and the propensity for CIRI, interventional and thrombolytic therapies have had limited clinical application [6]. Therefore, there is an urgent need to identify molecular targets that can inhibit neuronal apoptosis in the ischemic penumbra after CIRI, and to find effective drugs and/or methods for clinical application.

CIRI induces harmful cascades of pathological reactions, including energy metabolism disruptions, intracellular Ca^2+^ overload, blood-brain barrier destruction, excitatory amino acid receptor activation, inflammatory cytokine production and oxygen free-radical release [7–9]. These effects are interconnected and mutually reinforcing, forming a vicious cycle that ultimately leads to neuronal death. Apoptosis is the main cause of neuronal death in the ischemic penumbra, and thus is an important cause of CIRI [3]. However, the process of neuronal apoptosis is complex, and the signaling pathway remains unclear.

Endoplasmic reticulum stress (ERS) is known to contribute to neuronal apoptosis after CIRI [10]. The endoplasmic reticulum (ER) is the organelle responsible for the folding, post-translational modification, transport and secretion of lumen and membrane proteins [11]. When this dynamic environment becomes unbalanced and dysfunctional (for instance, due to ischemia or inflammation), the unfolded protein response occurs [12]. If the unfolded protein response persists or becomes too severe, toxic substances and unfolded proteins will accumulate in the ER, leading to ERS [13]. ERS activates downstream apoptotic pathways, induces noxious signals and impairs the function of tissues and organs [14]. However, the molecular triggers of ERS-induced apoptosis are not fully understood [15].

The Sigma-1 receptor (Sig-1R) is an important regulator of ERS. Sig-1R is widely distributed on the mitochondria-associated ER membrane (MAM) in tissues such as the brain and spinal cord [16, 17]. In the MAM, Sig-1R forms a complex with another ERS marker protein, GRP78 [18]. Upon ER Ca^2+^ depletion or ligand stimulation, ERS induces the dissociation of Sig-1R from GRP78 and promotes their translocation to the whole ER network to inhibit ERS-induced apoptosis [18]. Sig-1R has been reported to be a molecular chaperone in many biological processes, including stroke, retinal disease, neurodegenerative diseases and Huntington's disease [19–22]. Thus, the discovery of Sig-1R inducers may lead to breakthroughs in neuroprotective drug research.

Dexmedetomidine was introduced as a classic alpha 2 adrenergic receptor agonist for clinical perioperative use. It exerts dose-dependent sedative, hypnotic, analgesic and sympathetic blockade effects without significantly inhibiting respiration [23, 24]. In addition to its advantages in clinical anesthesia, dexmedetomidine has been reported to alleviate tissue ischemia-reperfusion injury [25]. The clinical use of dexmedetomidine has been demonstrated to suppress oxidative stress-induced damage, inhibit the inflammatory response, prevent apoptosis and reduce necrosis, thus protecting the heart, brain, kidney, liver, lung, gastrointestinal tract and other organs [25, 26]. Numerous studies have described a variety of ways that dexmedetomidine may alleviate CIRI, but there have been many contradictory results, so the mechanism whereby dexmedetomidine inhibits CIRI-induced apoptosis remains unclear.

In this study, we investigated whether the Sig-1R signaling pathway contributed to the protective (anti-apoptotic) effects of dexmedetomidine in CIRI, to provide a rationale for the widespread clinical use of dexmedetomidine.

## RESULTS

### Dexmedetomidine alleviated neurological dysfunction after CIRI

**Figure 1:**
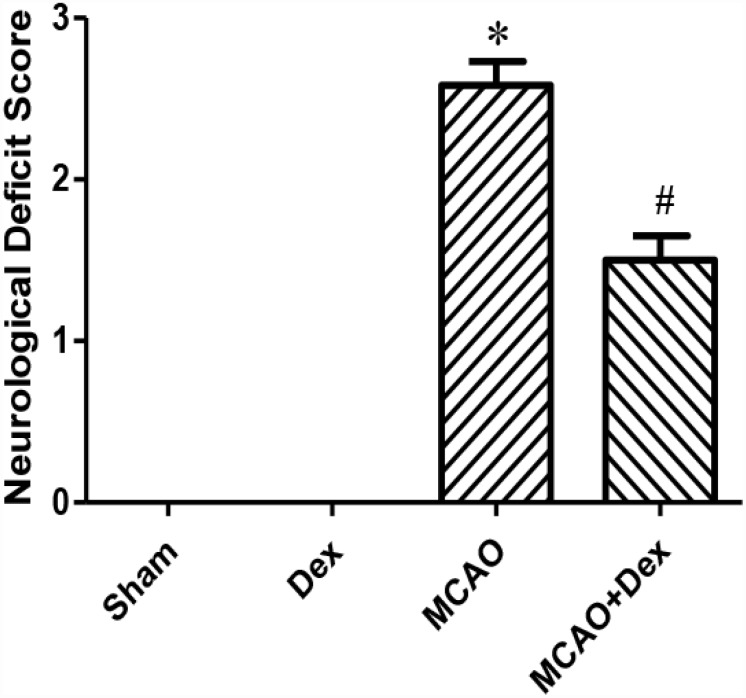
**Dexmedetomidine improved neurological function after CIRI.** Neurological deficit scores. Data are shown as the mean ± standard error of the mean (SEM), n = 12 per group. *P < 0.05 compared with the sham group; ^#^P < 0.05 compared with the MCAO group. Sham operation group, Sham group; dexmedetomidine administration group, Dex group; MCAO model control group, MCAO group; MCAO + dexmedetomidine administration group, MCAO + Dex group.

To study the effects of dexmedetomidine on CIRI, we established four groups of rats: a sham operation group, a dexmedetomidine treatment group, a middle cerebral artery occlusion (MCAO) model group and an MCAO + dexmedetomidine group. Twenty-four hours after inducing cerebral ischemia and reperfusion, we assessed the neurological deficits in each group by calculating the neurological function score (Figure 1). The preoperative neurological scores of all the groups were in the normal range. The neurological deficit scores in the dexmedetomidine group did not differ significantly from those in the sham group (P>0.05). However, the neurological deficit scores were significantly higher in the MCAO group than in the sham group (P<0.05). The neurological deficit scores in the MCAO + dexmedetomidine group were significantly lower than those in the MCAO group (P<0.05). These results demonstrated that dexmedetomidine had no obvious toxicity in normal rats, and ameliorated the neurological dysfunction caused by 24-hour CIRI.

### Dexmedetomidine attenuated the neuronal apoptosis induced by CIRI

**Figure 2:**
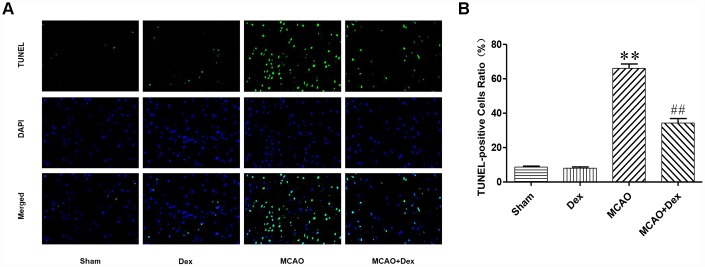
**Dexmedetomidine attenuated the neuronal apoptosis induced by CIRI.** (**A**) TUNEL staining was performed on slices from the cerebral ischemic penumbra. a. Sham operation (Sham) group, b. dexmedetomidine administration (Dex) group, c. MCAO model control (MCAO) group, d. MCAO + dexmedetomidine administration (MCAO + Dex) group. (**B**) Ratio of positive cells. Magnification is 400x. n = 6. Data are expressed as the mean ± SEM. *P < 0.05, **P < 0.01 compared with the sham group; ^#^P < 0.05, ^##^P < 0.01 compared with the MCAO group.

To investigate the molecular mechanism whereby dexmedetomidine protected against neurological dysfunction following CIRI, we used terminal deoxynucleotidyl transferase-mediated dUTP nick end labeling (TUNEL) staining to detect apoptosis in slices of the cerebral ischemic penumbra from each group (Figure 2). The number of apoptotic cells was significantly greater in the MCAO group than in the sham group, but this increase was attenuated in the MCAO + dexmedetomidine group (P<0.01). There was no significant difference in the rate of apoptosis between the dexmedetomidine group and the sham group (P>0.05). These results indicated that 24-hour CIRI significantly increased the rate of neuronal apoptosis, while dexmedetomidine exerted neuroprotective effects by inhibiting this neuronal apoptosis.

### Dexmedetomidine upregulated the expression of Sig-1R after CIRI

Sig-1R is widely distributed on the MAM and is an important contributor to the ERS response [16, 17].

**Figure 3:**
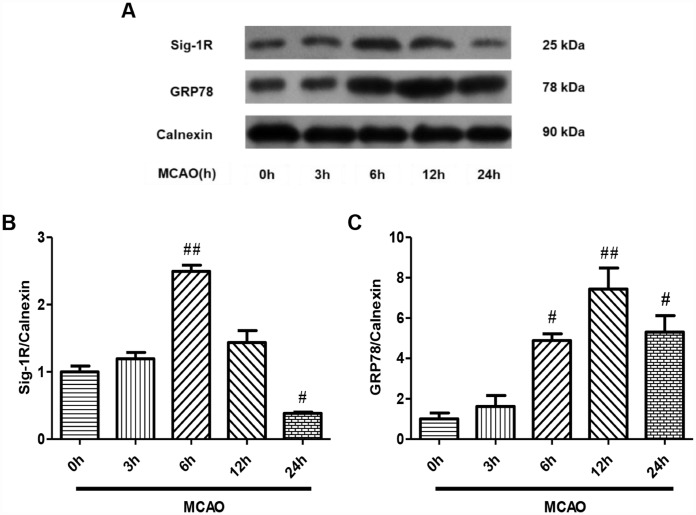
**Sig-1R and GRP78 protein expression on the ER after CIRI.** (**A**) The protein levels of Sig-1R and GRP78 were determined by Western blotting. (**B**) Sig-1R protein levels with increasing reperfusion times. (**C**) GRP78 protein levels with increasing reperfusion times. The results were normalized to the percentage of Calnexin expression. Data are shown as the mean ± SEM, n = 6 per group. ^#^P < 0.05, ^##^P < 0.01 compared with the MCAO 0-hour group.

ERS promotes the dissociation of Sig-1R from GRP78, freeing these proteins to inhibit ERS-induced apoptosis [18]. To investigate the involvement of Sig-1R in our CIRI model, we performed Western blotting to detect Sig-1R protein expression in ER-rich brain tissue lysates from each group. First, we examined the expression of Sig-1R after 0, 3, 6, 12 and 24 hours of ischemia-reperfusion in the MCAO model (Figure 3A). Sig-1R protein expression increased after 3 hours of reperfusion, increased significantly after 6 hours of reperfusion, and then decreased with increasing reperfusion times (P<0.05, Figure 3B). After 24 hours, the expression of Sig-1R was significantly lower than it was at 0 hours (P<0.05, Figure 3B). These results suggested that CIRI upregulated Sig-1R expression on the ER, and that Sig-1R was redistributed within cells as the reperfusion time increased.

**Figure 4:**
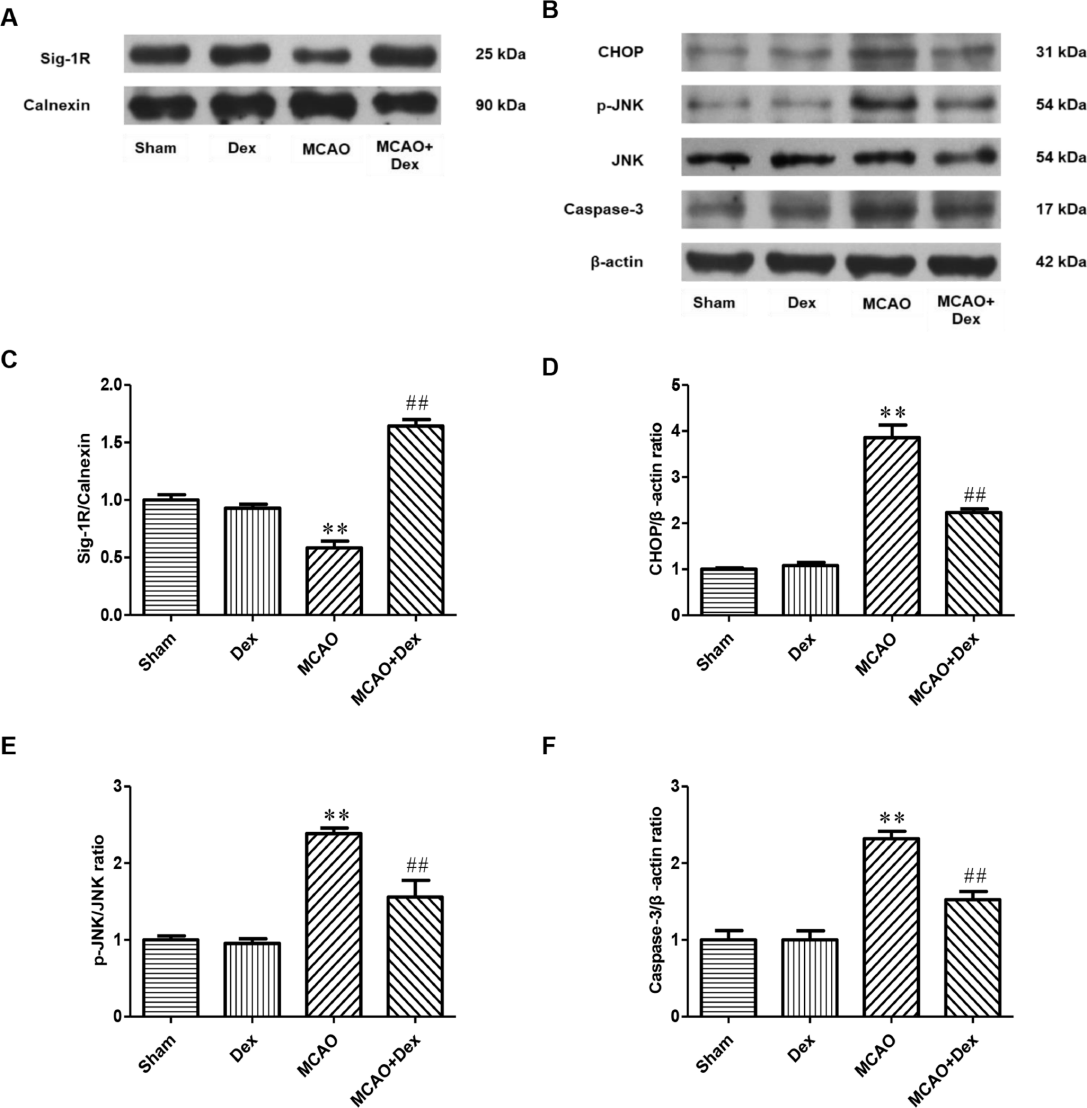
**The effects of dexmedetomidine on the expression of Sig-1R and ERS-induced apoptotic proteins after CIRI.** (**A**) The protein levels of Sig-1R in the cerebral ischemic penumbra were determined by Western blotting. (**B**) The levels of ERS-induced apoptotic proteins (CHOP, p-JNK/JNK, Caspase-3) in the cerebral ischemic penumbra were determined by Western blotting. (**C**) Sig-1R protein expression. (**D**) CHOP protein expression. (**E**) p-JNK/JNK protein expression. (**F**) Caspase-3 protein expression. The results were normalized to the percentage of Calnexin or β-actin expression. Data are shown as the mean ± SEM, n = 6 per group. *P < 0.05, **P < 0.01 compared with the sham group; ^#^P < 0.05, ^##^P < 0.01 compared with the MCAO group. Sham operation group, Sham group; dexmedetomidine administration group, Dex group; MCAO model control group, MCAO group; MCAO + dexmedetomidine administration group, MCAO + Dex group.

Subsequently, we examined the expression changes of Sig-1R in brain tissues from the four groups of rats after 24 hours of ischemia-reperfusion. Sig-1R expression was significantly lower in the MCAO group than in the sham group, but this decrease was significantly attenuated in the MCAO + dexmedetomidine group (P<0.05, Figure 4A, 4C). These results suggested that dexmedetomidine upregulated the expression of Sig-1R in brain tissues after 24-hour CIRI.

### The upregulation of Sig-1R by dexmedetomidine may have inhibited apoptotic signaling in the ER

To determine the effects of Sig-1R activation on the downstream ERS signaling pathway after CIRI, we examined the expression of the ERS marker protein GRP78 in ER-rich brain tissue lysates at different ischemia-reperfusion time points by Western blotting (Figure 3A). GRP78 protein expression increased slightly after 3 hours of reperfusion, increased significantly after 6 hours of reperfusion, reached a peak at 12 hours, and then decreased at 24 hours (P<0.05, Figure 3C). Thus, the protein expression trend of GRP78 was basically consistent with that of Sig-1R.

To determine the effects of dexmedetomidine on the ERS signaling pathway involving Sig-1R after CIRI, we detected the expression of ERS marker proteins (C/EBP homologous protein [CHOP], c-Jun N-terminal kinase [JNK] and Caspase-3) in brain tissues by Western blotting (Figure 4B). CHOP and Caspase-3 levels were significantly higher in the MCAO group than in the sham group, as was the ratio of phosphorylated JNK to total JNK (P<0.01, Figure 4D–4F). These increases were significantly attenuated in the MCAO + dexmedetomidine group (P<0.01, Figure 4D–4F). These results suggested that 24-hour CIRI activated ERS and promoted neuronal apoptosis, while dexmedetomidine inhibited neuronal apoptosis through this pathway.

**Figure 5:**
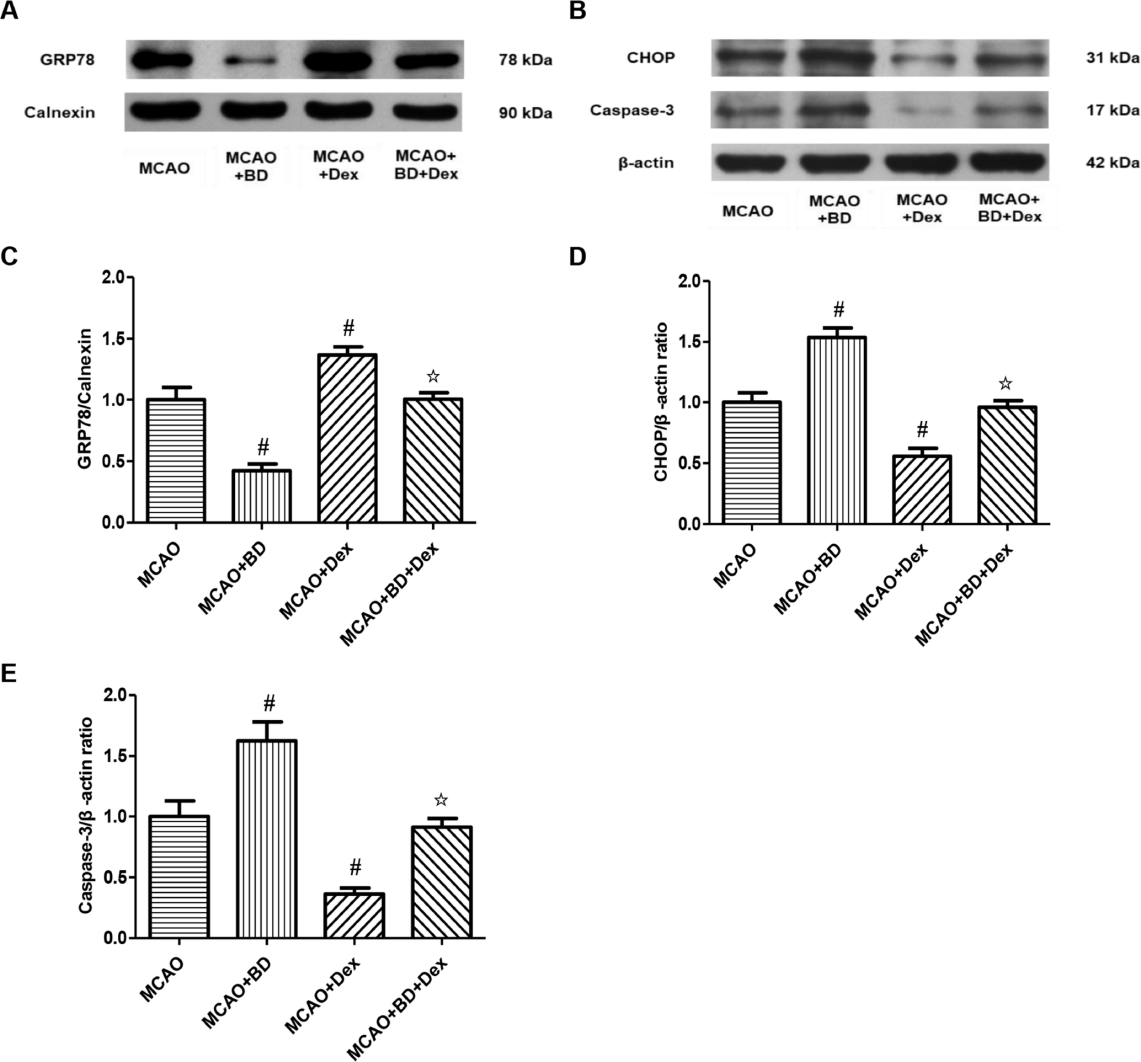
**The effects of dexmedetomidine on the ERS signaling pathway involving Sig-1R after CIRI.** (**A**) GRP78 protein levels on the ER were determined by Western blotting. (**B**) The ERS-induced apoptotic proteins (CHOP, Caspase-3) from the cerebral ischemic penumbra were determined by Western blotting. (**C**) GRP78 protein levels. (**D**) CHOP protein levels. (**E**) Caspase-3 protein levels. The results were normalized to the percentage of Calnexin or β-actin expression. Data are shown as the mean ± SEM, n = 6 per group. ^#^P < 0.05, ^##^P < 0.01 compared with the MCAO group; ^☆^P < 0.05, ^☆☆^P < 0.01 compared with the MCAO + dexmedetomidine group. MCAO + BD1063 group, MCAO + BD group; MCAO + dexmedetomidine group, MCAO + Dex group; MCAO + BD1063 + dexmedetomidine group, MCAO + BD + Dex group.

To confirm the effects of dexmedetomidine on the Sig-1R-associated ERS signaling pathway, we treated rats with a Sig-1R-specific inhibitor (BD1063) 30 minutes before performing the MCAO operation, and subsequently detected the changes in GRP78 expression in ER-rich brain tissue lysates (Figure 5A). Compared with the MCAO group, the expression of GRP78 on the ER was significantly reduced in the MCAO + BD1063 group, while it was significantly elevated in the MCAO + dexmedetomidine group (P<0.05, Figure 5C). GRP78 expression was significantly lower in the MCAO + BD1063 + dexmedetomidine group than in the MCAO + dexmedetomidine group (P<0.05, Figure 5C). These results suggested that the Sig-1R-specific inhibitor significantly reduced GRP78 expression in brain tissues after 24-hour CIRI, and also prevented the upregulation of GRP78 by dexmedetomidine to a certain extent.

We also examined the protein levels of the ERS-induced apoptotic markers CHOP and Caspase-3 in brain tissues, and found that they were significantly higher in the MCAO + BD1063 group than in the MCAO group, but were significantly lower in the MCAO + dexmedetomidine group (P<0.05, Figure 5B, 5D, 5E). CHOP and Caspase-3 levels were significantly higher in the MCAO + BD1063 + dexmedetomidine group than in the MCAO + dexmedetomidine group (P<0.05, Figure 5B, 5D, 5E). These results suggested that dexmedetomidine may at least partially inhibit ERS-induced apoptosis by activating Sig-1R in brain tissues, thus attenuating brain damage after 24 hours of ischemia-reperfusion.

## DISCUSSION

Ischemic stroke is a high-incidence disease of the nervous system, and is the main cause of disability and death in adults. Although the duration of ischemia is a decisive determinant of the occurrence of secondary injuries, the effects of reperfusion injury should not be underestimated. Ischemia-reperfusion injuries occur in a variety of brain diseases, the most common of which is acute ischemic stroke [3]. Ischemic apoptosis is the main form of neuronal loss after CIRI, and is the primary cause of recurrent ischemic stroke [3].

It is presently believed that CIRI disrupts the “cell balance system” by disturbing the intracellular calcium homeostasis, inducing inflammatory reactions, promoting oxygen free-radical damage, stimulating the release of excitatory amino acids, impairing mitochondrial function, promoting cell autophagy, activating the apoptotic pathway and so on [1, 27]. These disturbances overlap with one another and form a vicious cycle that eventually leads to apoptosis or necrosis. Thus, each of these mechanisms is a potential therapeutic target in CIRI. Certain anti-inflammatory agents, oxidative stress inhibitors and apoptotic inhibitors have exhibited some benefits *in vitro* or *in vivo* in the treatment of CIRI, but clinical trials have not had the desired results [3]. Therefore, there is an imminent need to identify new drugs with different targets to achieve neuroprotective effects.

A large amount of evidence indicates that the recovery of blood flow after an ischemic stroke can lead to the formation of an ischemic penumbra in the arterial occlusion blood supply area, which is a region of great concern in terms of brain protection [2]. The cell damage induced by ischemia-reperfusion injury includes both cell necrosis in the ischemic center and apoptosis in the ischemic penumbra [3]. In the ischemic center, the blood flow drops sharply and cells die within a few minutes of ischemia and hypoxia. The nerve cells in the central area of ischemia are necrotic, and this damage is irreversible. However, due to collateral blood flow, the neurons in the ischemic penumbra are not completely blocked from their energy supply, and thus are buffered from ischemic tissue damage. These neurons still have relatively complete structures and metabolic capacities, and may survive for hours or even days, but will undergo apoptosis under further damage stimulation. We found that CIRI led to neurological dysfunction in rats and significantly increased the rate of neuronal apoptosis in the ischemic penumbra. However, apoptosis is a reversible form of programmed cell death, so the timely inhibition of neuronal apoptosis in the ischemic penumbra can prevent the further development of CIRI and achieve therapeutic effects [28]. Thus, inhibiting neuronal apoptosis in the ischemic penumbra is the main strategy for stroke rehabilitation.

Although mitochondrial pathways (including death receptor pathways) have been proposed to be the core mechanisms of apoptosis [29], the ER is structurally and functionally coupled with various organelles (including mitochondria) and also contributes to various apoptotic pathways [10]. CIRI can cause ER dysfunction, which induces a series of adaptive responses collectively referred to as ERS [30]. An excessive intensity or duration of ERS will activate apoptotic pathways [14]; thus, the ERS pathway is of great significance to the study of post-stroke rehabilitation [30].

Three ER transmembrane receptors are known to be involved in the ERS response: PKR-like ER kinase (PERK), inositol requiring protein 1 (IRE1) and activating transcription factor 6 (ATF6). Under normal conditions, the ER chaperone GRP78/BIP binds to these receptors and inhibits their function. However, ERS increases the expression of GRP78 and induces its dissociation from these transmembrane proteins. GRP78 can then bind to unfolded/misfolded proteins to facilitate their refolding and re-modification. This process repairs ER function and promotes tolerance to adverse factors. At the same time, the signaling pathways involving PERK, IRE1 and ATF6 are activated, and these pathways induce the expression of CHOP, JNK, Caspase-3 and other related molecules, ultimately promoting apoptosis [31]. The transcription factor CHOP is an important executor of ERS-induced apoptosis [32]. CHOP protein expression is low under non-stress condition, but increases significantly under ERS, thereby activating the apoptotic pathway [26]. In the ischemic penumbra, the balance between GRP78 and CHOP determines whether cells will survive or undergo apoptosis. We found that GRP78 was initially activated and was subsequently downregulated with increasing reperfusion times after 24-hour CIRI. Combined with the previous research basis, this suggests that CIRI may inhibit neuron apoptosis by up regulating GRP78 at the early stage of reperfusion, and its protective function decreased with the ischemia-reperfusion time. After 24 hours of ischemia-reperfusion injury, the expression of the ER apoptotic protein CHOP was significantly upregulated, along with p-JNK and Caspase-3, suggesting that CIRI could activate ERS signaling to promote apoptosis.

Sig-1R, a chaperone protein of the ER, is most abundant in the MAM, and normally complexes with GRP78/BIP [18]. However, under ERS, Sig-1R dissociates from GRP78 and is redistributed within the cell. Sig-1R has been reported to regulate retinal cell pressure [20], to inhibit the neuropathic pain caused by partial sciatic nerve transection in mice [33], to lessen the severity of depressive disorder and to have neurorestorative effects in Parkinson's disease [34]. Sig-1R exerts neuroprotective effects by regulating Ca^2+^ homeostasis and preventing glutamate toxicity and neuroinflammation [34]. We found that Sig-1R expression on the ER was significantly upregulated in the early stage of CIRI, and decreased with increasing reperfusion times, consistent with the expression trend of GRP78. These results suggest that the activation of Sig-1R and GRP78 increased gradually with the prolongation of CIRI, and decreased at 24 hours of reperfusion, while the expression of other ERS apoptotic proteins increased significantly at 24 hours of reperfusion. Combined with previous studies, the results suggest that CIRI may activate two proteins (Sig-1R and GRP78), and promote intracellular redistribution, thereby exerting brain protection. With the prolongation of CIRI and the aggravation of brain injury, the ERS apoptotic pathway is activated 24 hours of ischemia, which promotes the occurrence of CIRI. These results suggested that 24-hour CIRI inhibited ERS-induced apoptosis by promoting the activation of Sig-1R and GRP78. Thus, the ERS pathway involving Sig-1R is a key component of the apoptotic cascade after 24-hour CIRI, and could be a new target for the prevention and treatment of ischemic stroke.

Dexmedetomidine is widely used for perioperative anesthesia, clinical sedation and analgesia, but basic and clinical studies have also demonstrated that dexmedetomidine can protect various organs from ischemia-reperfusion injury (including the brain in CIRI) [25]. Dexmedetomidine suppressed cerebral-ischemia-induced oxidative stress and apoptosis by inhibiting the activation of the TRPM2 and TRPV1 channels in neurons [35]. Pretreatment with dexmedetomidine exerted neuroprotective effects in a model of cerebral ischemia-reperfusion by inhibiting the TLR4/NF-κB pathway and reducing inflammatory damage [36]. Activation of mitochondrial ATP channels may have contributed to the protective effects of dexmedetomidine against focal CIRI in rats [37]. Dexmedetomidine was found to counteract the apoptotic damage induced by cerebral ischemia-reperfusion through an intrinsic mitochondrial pathway [38]. The dexmedetomidine-induced phosphorylation of cyclic AMP response-element binding protein enhanced ischemic tolerance in a mouse model of spinal cord ischemia-reperfusion [39]. Dexmedetomidine inhibited neuronal apoptosis by suppressing the HIF-1α pathway, and also inhibited neuronal autophagy in CIRI by upregulating HIF-1α [40, 41]. *In vitro*, dexmedetomidine alleviated the mitochondrial apoptosis induced by hypoxia/reoxygenation in hippocampal neurons by activating HIF-1a/p53 signal transduction [42]. Thus, dexmedetomidine exerts neuroprotective activity by inhibiting inflammation, oxidative reactions, autophagy and apoptosis through a variety of signaling pathways.

A recent study revealed that dexmedetomidine attenuated CIRI by inhibiting ERS-induced apoptosis [26]. In agreement with these findings, our results suggested that dexmedetomidine improved neurological function and inhibited CIRI-induced neuronal apoptosis. We also found that dexmedetomidine upregulated Sig-1R and GRP78 expression after 24 hours of CIRI and inhibited the ERS-induced expression of pro-apoptotic proteins (CHOP, p-JNK and Caspase-3). A Sig-1R-specific inhibitor suppressed the expression of GRP78 after CIRI and also prevented its upregulation by dexmedetomidine to a certain extent. Furthermore, the inhibitor partially reversed the inhibitory effects of dexmedetomidine on CHOP and Caspase-3 expression after CIRI. These results suggested that dexmedetomidine at least partially inhibited neuronal apoptosis in the ischemic penumbra and attenuated brain damage after 24 hours of ischemia-reperfusion by upregulating the expression of Sig-1R.

In conclusion, this study elucidated the involvement of the Sig-1R-associated ERS-induced apoptotic pathway in CIRI. By preliminarily exploring the protective mechanism of dexmedetomidine in CIRI, we have provided a good theoretical and experimental basis for its clinical application in ischemic stroke. However, the possible regulation relationship between Dex treatment and Sig-1R is still in the initial stage of research, and other pathways involved have not been studied. Therefore, more research is needed to reveal the possible mechanisms, fill the gaps, and provide effective drug targets for the clinical treatment of CIRI.

## MATERIALS AND METHODS

### Experimental animals

In total, 102 healthy specific-pathogen-free adult male Sprague Dawley rats (age: 8-12 weeks, weight: 220-260 g) were provided by Beijing HFK Bioscience Co., Ltd. (Institute of Medical Laboratory Animals, Chinese Academy of Medical Sciences)**.** The rats were acclimated to the laboratory for one week before the experiment. The animals were housed four per cage and were free to ingest standard feed and water. The room temperature was set to 22 ± 2 °C, the relative humidity was set to 50-60%, and a 12-hour light-dark cycle was used. The room was regularly ventilated, the water and feed were exchanged and the cushions were replaced to keep the animals healthy. All the animal experiments were approved by the Experimental Animal Ethics Committee of the Tianjin Central Hospital of Gynecology Obstetrics and were performed in strict accordance with the guidelines of the National Laboratory Animal Ethics Regulations.

### Animal model preparation

The rats were fasted for 12 hours before the operation, but were allowed access to water. The MCAO model in rats was established by thread embolization according to the Zea-Longa method [43]. The rats were anesthetized by intraperitoneal injection of a 10% chloral hydrate solution (300 mg/kg) and fixed in the supine position. The total neck and internal and external carotid arteries along the midline of the neck were carefully separated. The proximal common end of the common carotid artery and the distal end of the external carotid artery were ligated. Then, a prepared nylon thread bolt was slowly inserted into the internal carotid artery, stopped advancing when encountering slight resistance, and the bolt was fixed with a reserved line. After the blood flow was successfully blocked for two hours, the bolt was pulled out and reperfusion was allowed to proceed for 24 hours. The wound was stitched layer-by-layer at the end of the operation. During the procedure, the ambient temperature was maintained at 37 ± 0.5 °C, and the rectal temperature, respiration rate and heart rate were monitored. After the rats were awakened, they were returned to the animal house for feeding and observation.

### Grouping and treatment of the experimental animals

Our experiments were divided into three parts. Firstly, to elucidate the possible protective mechanism of dexmedetomidine in a model of CIRI, we randomly divided the rats into four groups: the sham operation group, the dexmedetomidine administration group (dexmedetomidine was purchased from Jiangsu Enhua Pharmaceutical Co., Ltd., National Drug Standard H20110086), the MCAO model control group, and the MCAO + dexmedetomidine administration group. TUNEL staining and Western blotting were performed after the neurological deficit score was determined. Secondly, to observe the effects of the ERS pathway involving Sig-1R on CIRI, we subjected rats to ischemia for two hours and randomly sampled their brain tissues at five time points of reperfusion (0, 3, 6, 12 and 24 hours) for Western blotting. Finally, to verify that the ERS pathway involving Sig-1R was responsible for the protective effects of dexmedetomidine in CIRI, we obtained a specific inhibitor of Sig-1R (BD1063, MedChemExpress; cat. no. HY-18101A) and randomly divided the rats into four groups: the MCAO group, the MCAO + BD1063 group, the MCAO + dexmedetomidine group, and the MCAO + BD1063 + dexmedetomidine group. Western blotting was performed after the neurological deficit score was determined.

In the above groupings, the sham group underwent the same anesthesia procedure and operation as the MCAO group, but a thread was not inserted into the middle cerebral artery. For the dexmedetomidine group, dexmedetomidine was intravenously administered at a loading dose of 1 μg/kg at the very beginning of the surgery, and was then administered at 0.05 μg/kg/min for the next two hours [26, 44]. The other operations in the dexmedetomidine group were the same as in the sham group. In the MCAO model control group, the middle cerebral artery thread occlusion method was used for two hours. Then, the thread thrombus was pulled out and reperfusion was allowed to proceed for 24 hours. In the MCAO + dexmedetomidine group, the dexmedetomidine treatment was the same as in the dexmedetomidine group, and the rest of the operation was the same as in the MCAO group. In the MCAO + BD1063 group, BD1063 (3 mg/kg) was injected subcutaneously 30 minutes before the operation, and the rest of the operation was the same as in the MCAO group [45]. In the MCAO + BD1063 + dexmedetomidine group, BD1063 was injected subcutaneously 30 minutes before the operation, dexmedetomidine was intravenously administered at the beginning of the surgery, and the rest of the operation was the same as in the MCAO group. The sham and MCAO groups were administered the same amount of normal saline. The rats were placed in a clean environment after the surgery, and were free to eat and drink under the same conditions previously described.

### Neurological deficit score

The neurobehavioral score was scored by a researcher who was blinded to the experimental groups. After the rats were subjected to 24 hours of CIRI, the neurological function of each rat was scored according to Zea-Longa’s five-point scoring system [43]: 0 points, the rat had no neurological symptoms; 1 point, the rat could not fully extend its contralateral forepaw when lifting its tail; 2 points, the rat turned to the opposite side of the operation while walking; 4 points, the rat could not walk on its own or lost consciousness. A neurological deficit score of 2 to 3 was used as the success criterion for establishing the MCAO model. The rats that did not meet this criterion were excluded, along with those that underwent subarachnoid hemorrhaging or death within 24 hours. Additional rats were randomly sampled under the same conditions and were subjected to the same experimental procedures to ensure that each group contained an adequate number of rats.

### Material and specimen processing

After 24 hours of CIRI, six rats in each group were randomly selected for the TUNEL assay or Western blotting. The rats were intraperitoneally anesthetized with a 10% chloral hydrate solution (300 mg/kg) and fixed on the operating table. The anterior cardiac region was fully exposed, the left ventricle was punctured with a perfusion needle from the apex, and the needle was fixed. The right auricle was cut and the tissue was continuously perfused with 0.9% saline until no bloody liquid was observed. Then, the perfusion solution was replaced with 4% paraformaldehyde until the limbs of the rats were stiff. The cerebral cortex on the side of the CIRI was separated. The penumbra of the cerebral cortex on the side of the CIRI was isolated according to previously published methods [26, 46]. We initially cut about 2 mm from the outside of the sagittal suture through the right hemisphere longitudinally (from top to bottom). Lateral diagonal cuts were then made at approximately the “2 o’clock” position, and the penumbra was separated from the core for further experimentation. The isolated penumbra of the cerebral cortex was fixed at 4 °C for the TUNEL experiment. After cardiac perfusion with ice-cold saline, the brain was removed and the penumbra of the cerebral cortex on the side of the CIRI was separated on ice. The penumbra of the cerebral cortex was stored in a liquid nitrogen tank until Western blotting.

### Detection of apoptosis by TUNEL staining

The fixed samples of the cerebral ischemic penumbra were embedded in paraffin and sliced into 5-micron sections for TUNEL staining. The experiment was carried out strictly according to the instructions of the TUNEL staining kit (Roche Diagnostics Corp., Indianapolis, IN, USA; Cat. No. 11684817910). Under 400x fluorescence microscopy, four non-overlapping visual fields were randomly selected, and the percentage of the apoptotic cell index (ACI%, the number of apoptotic cells/the total number of apoptotic cells × 100%) was calculated.

### Western blotting experiment

Protein isolation and Western blotting were performed as previously described [18]. In short, the cryopreserved rat brain tissue samples from the CIRI side were homogenized with lysis buffer on ice, and the lysate was left on ice until full lysis was achieved. The tissue homogenate was centrifuged at 12,000 x *g* for 30 minutes at 4 °C. The supernatant was the total cell lysate, and an appropriate amount was collected for Western blotting. The remaining ER-rich cerebral ischemic penumbra was centrifuged (800 × *g*) for 10 minutes at 4 °C, and the resulting supernatant was centrifuged (10,000 × *g*) for 20 minutes at 4 °C. The new supernatant was collected at 4 °C and centrifuged at 100,000 × *g* for 60 minutes, yielding an ER-rich precipitate. The precipitate was added to a lysis buffer containing 1% Triton X-100, and an appropriate amount was collected for Western blot analysis.

The protein concentration was determined by the bicinchoninic acid method. A sodium dodecyl sulfate polyacrylamide gel was prepared, and 30 μg of protein was loaded per well. The proteins were electrophoretically separated and transferred to a polyvinylidene fluoride membrane. The membrane was washed thoroughly and blocked with 5% skim milk powder for two hours. The membrane was cut into strips and incubated overnight at 4 °C with one of the following primary antibodies: Sig-1R (1:1000; Proteintech Group, Wuhan, China; cat. no. 15168-1-AP), GRP78 (1:3000; Abcam, Cambridge, UK; cat. no. ab108613), p-JNK (1:5000; Abcam, Cambridge, UK; cat. no. ab124956), JNK (1:1000; Abcam, Cambridge, UK; cat. no. ab179461), CHOP (1:1000; Abcam, Cambridge, UK; cat. no. ab11419), Caspase-3 (1:2000; Abcam, Cambridge, UK; cat. no. ab184787). β-actin (1:3000; Proteintech Group, Wuhan, China; cat. no. 20536-1-AP) and Calnexin (1:1000; Abcam, Cambridge, UK; cat. no. ab133615) were used as internal references. After the membrane was thoroughly washed, a horseradish peroxidase-labeled secondary antibody (1:1000; Solarbio, Beijing, China; cat. no. SE134 or SE131) was applied for two hours at room temperature prior to enhanced chemiluminescence development. Image J software was used for image analysis. The relative expression of the target strip was expressed as the ratio of the gray value of the strip to the gray value of the internal reference strip.

### Statistical analysis

The Data were expressed as mean ± standard deviation (SD). The statistical significance of differences between groups was obtained using one-way analysis of variance (ANOVA) for multiple comparisons. The statistical analyses were conducted with GraphPad Prism 5.0 software. P < 0.05 was considered statistically significant.
